# Supplementary material for: Source Areas as a Key Factor Contributing to the Recovery Time of Controlled Feral Pigeon (Columba livia var. domestica) Colonies in Low-Density Urban Locations
Source: Animals (Basel). 2022 Apr 19;12(9):1056. doi: 10.3390/ani12091056 (PMC9101645; doi:10.3390/ani12091056)
Supplement: Supplementary file 1 [file animals-12-01056-s001.zip › animals-1554328-supplementary.pdf]

animals

MDPI

Supplementary materials

Source Areas as a Key Factor Contributing to the Recovery Time of Controlled Feral Pigeon (*Columbia livia* var. *domestica*) Colonies in Low-Density Urban Locations

Miguel Ángel Farfán Aguilar [1,2,*], Jesús Duarte [3] and Francisco Díaz-Ruiz [1,2]

[1] Departamento de Biología Animal, Facultad de Ciencias, Universidad de Málaga, Campus de Teatinos, 29071 Málaga, Spain; pacodi1480@hotmail.com
[2] Instituto IBYDA, Centro de Experimentación Grice-Hutchinson, Loma de San Julián 2, Barriada de San Julián, 29004 Málaga, Spain
[3] Ofitecma Marbella S.L., Av. Ramón y Cajal 17, 29601 Marbella, Spain; jddofitecma@gmail.com
* Correspondence: mafarfan@uma.es

Citation: Farfán Aguilar, M.Á.; Duarte, J.; Díaz-Ruiz, F. Source Areas as a Key Factor Contributing to the Recovery Time of Controlled Feral Pigeon (*Columbia livia* var. *domestica*) Colonies in Low-Density Urban Locations. *Animals* **2022**, *12*, 1056. https://doi.org/10.3390/ani12091056

Academic Editors: Jukka Jokimäki and Reuven Yosef

Received: 29 December 2021
Accepted: 12 April 2022
Published: 19 April 2022

Publisher's Note: MDPI stays neutral with regard to jurisdictional claims in published maps and institutional affiliations.

Copyright: © 2022 by the authors. Licensee MDPI, Basel, Switzerland. This article is an open access article distributed under the terms and conditions of the Creative Commons Attribution (CC BY) license (http://creativecommons.org/licenses/by/4.0/).

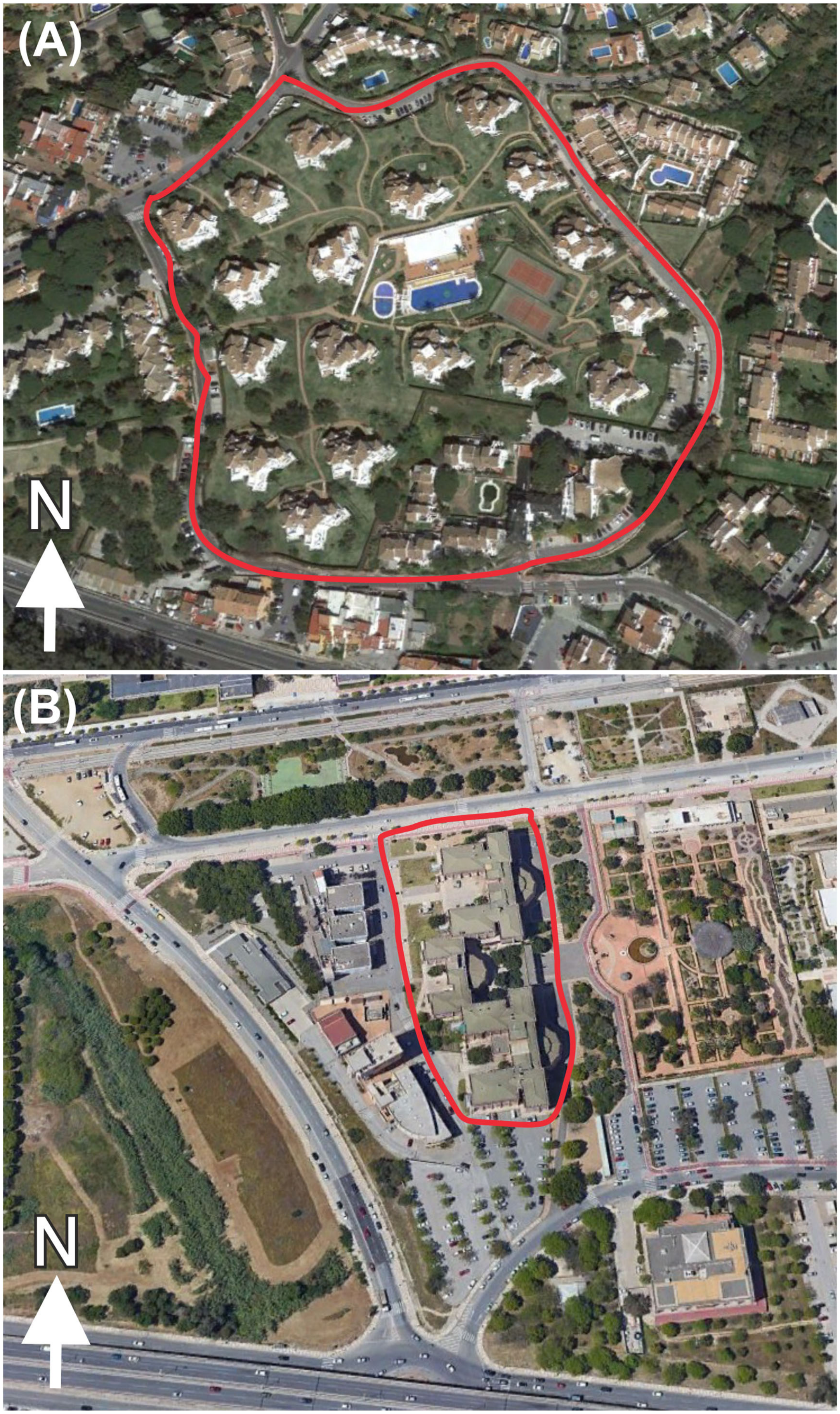
(A)
N
(B)
N

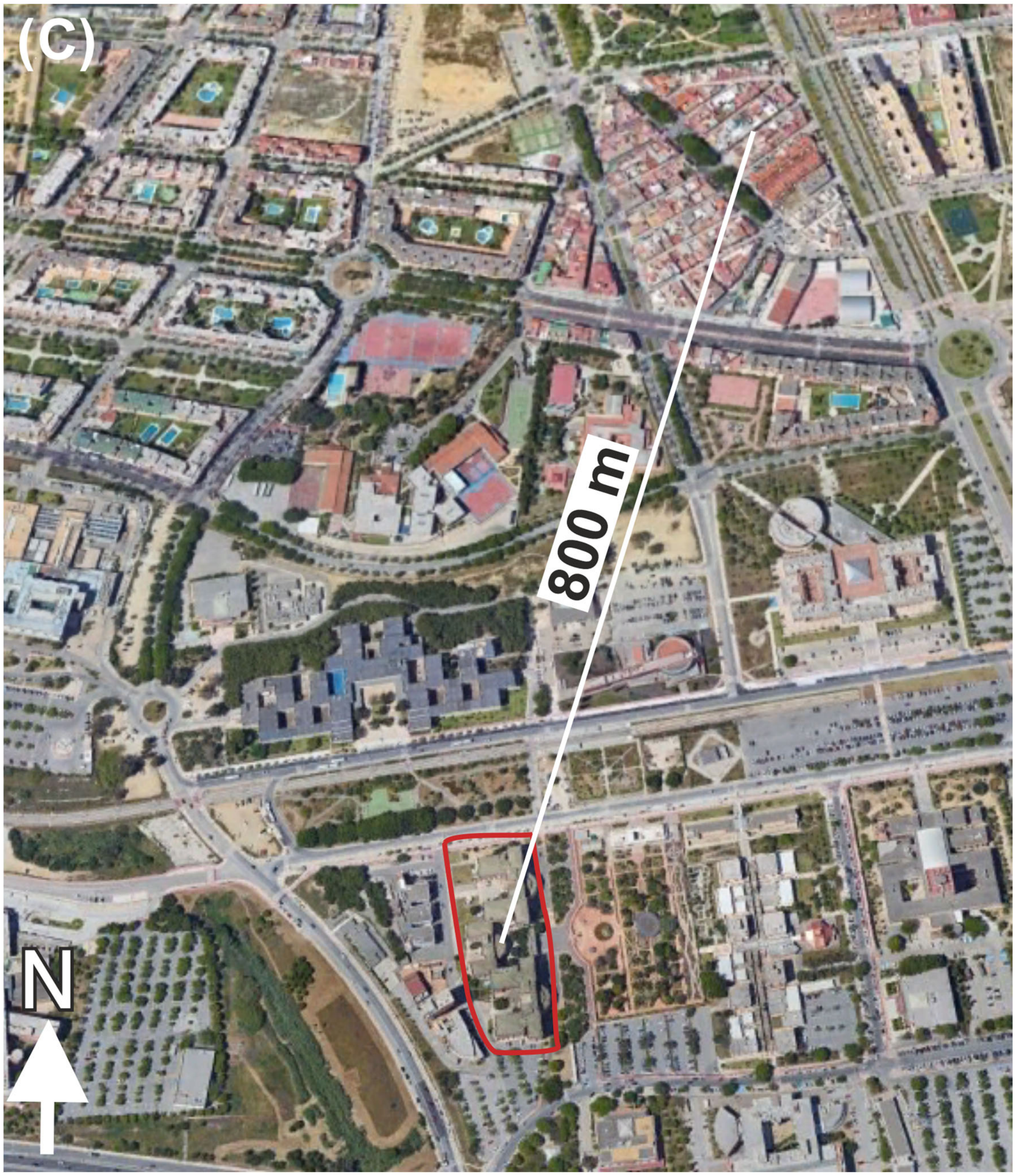

Figure S1. Examples of urban areas in which we have carried out Pigeon control programs (marked with red line). Residential area (**A**) and isolated building (**B**) surrounded by green areas, gardens, and wooded patches. (**C**) Example of estimating the distance between the building under control (marked with red line) and the closest Pigeon colony. Basemap source: Google Earth (https://www.google.es/intl/es/earth/index.html accessed on 1 December 2021). This figure was prepared in CorelDRAW 2020 (https://www.coreldraw.com/la/product/coreldraw/ accessed on 1 December 2021).

Table S1. Features of the buildings included in this study. Map_ID: location of the buildings in the study area (see Fig. 1). Date: Years in which the control campaigns were carried out in each building during the study period. Season: season of the year where the control programme is carried out. Spr: spring; Sum: summer; Aut: autumn; Win: winter. Days: time elapsed between successive control campaigns. Distance: the distance to the closest area in which Feral Pigeons were present. Pigeons B: number of Feral Pigeons before control campaign. Pigeons E: number of Feral Pigeons at the end of the control campaign.

Building	Map_ID	Date	Season	Days	Distance (m)	Pigeons B	Pigeons E
**1**	1	2007	Spr-Sum-Aut-Win	160	1600	40	0
**1**	1	2009	Spr-Sum	553	1600	30	2
**2**	2	2004-2005	Spr-Sum-Aut-Win	214	50	7	1
**3**	2	2005-2006	Sum-Aut	50	50	12	3
**4**	2	2004-2005	Spr-Sum-Aut-Win	54	50	46	0
**5**	2	2004-2009	Sum-Aut-Win	1132	871	29	0
**6**	3	2003	Spr-Sum	367	1100	37	0
**6**	3	2007	Spr-Sum	1487	1100	41	0
**6**	3	2010	Spr-Sum	1265	1100	33	3
**7**	4	2007-2009	Sum	349	300	11	0
**8**	4	2007-2010	Spr-Sum-Aut-Win	820	1000	18	6
**9**	5	2006-2009	Spr-Sum-Aut	531	1400	51	0
**10**	6	2008	Sum-Aut-Win	74	160	10	0
**10**	6	2009	Sum-Aut	77	160	14	8
**11**	7	2003	Spr-Sum	213	796	5	1
**11**	7	2010	Spr-Sum-Aut	3072	2000	48	4
